# Role of Morphology on Zinc Oxide Nanostructures for Efficient Photoelectrochemical Activity and Hydrogen Production

**DOI:** 10.3390/ma17205135

**Published:** 2024-10-21

**Authors:** Ahmad Fallatah, Mohammed Kuku, Laila Alqahtani, Almqdad Bubshait, Noha S. Almutairi, Sonal Padalkar, Abdullah M. Alotaibi

**Affiliations:** 1 Future Mobility Institute, King Abdulaziz City for Science and Technology, Riyadh 11442, Saudi Arabia; afallatah@kacst.edu.sa; 2 Desalination Technologies Institute, King Abdulaziz City for Science and Technology, Riyadh 11442, Saudi Arabia; 3 Department of Mechanical Engineering, College of Engineering and Computer Science, Jazan University, Jazan 45142, Saudi Arabia; mkuku@jazanu.edu.sa; 4 Advance Materials Institute, King Abdulaziz City for Science and Technology, Riyadh 11442, Saudi Arabia; lmalqahtani@kacst.gov.sa (L.A.); nalmutairi@kacst.gov.sa (N.S.A.); 5 Hydrogen Technologies Institute, King Abdulaziz City for Science and Technology, Riyadh 11442, Saudi Arabia; abubshait@kacst.gov.sa; 6 Department of Mechanical Engineering, Iowa State University, Ames, IA 50011, USA

**Keywords:** zinc oxide, electrodeposition, morphology, water splitting, nanostructure, photocatalysis

## Abstract

Energy generation today heavily relies on the field of photocatalysis, with many conventional energy generation strategies now superseded by the conversion of solar energy into chemical or thermal energy for a variety of energy-related applications. Global warming has pointed to the urgent necessity of moving away from non-renewable energy sources, with a resulting emphasis on creating the best photocatalysts for effective solar conversion by investigating a variety of material systems and material combinations. The present study explores the influence of morphological changes on the photoelectrochemical activity of zinc oxide nanostructures by exploiting electrodeposition and capping agents to control the growth rates of different ZnO facets and obtain well-defined nanostructures and orientations. A zinc nitrate (Zn (NO_3_)_2_) bath was used to electrodeposit ZnO nanostructures on an indium tin oxide glass (ITO) substrate at 70 °C with an applied potential of −1.0 V. Ethylenediamine (EDA) or ammonium fluoride (NH_4_F) were added as capping agents to the zinc nitrate bath. Extensive evaluation and characterization of the photoelectrochemical (PEC) capabilities of the resulting morphology-controlled zinc oxide nanostructures confirmed that altering the ZnO morphology can have positive impacts on PEC properties.

## 1. Introduction

Carbon emissions from the combustion of fossil fuels are the most significant cause of global warming and its effects, which have started to appear in recent years [1,2,3]. Consequently, the creation of clean, efficient, sustainable, and low-carbon energy sources is essential to reduce global warming and achieve carbon neutrality [4]. Hydrogen is one viable source of clean energy, as it emits no carbon dioxide when consumed. In fact, hydrogen fuel cell technology is now viewed as one of the best ways to address the energy crisis in the future [5]. However, industrial hydrogen production currently depends on fossil fuels and industrial waste products and, therefore, produces significant carbon emissions, with 80% of present-day hydrogen production coming from coal and natural gas as raw materials [6]. One promising strategy for creating green hydrogen is to use solar power for water splitting to generate hydrogen with zero carbon emissions. Furthermore, the conversion of solar energy into chemical energy, such as hydrogen, methanol, or ammonia, can also aid in resolving the storage issue created by intermittent solar energy. Using solar energy would, therefore, enable a wider range of hydrogen uses, including powering electric cars and peak shaving on the power grid [7].

Water splitting is an efficient process because it produces hydrogen from water, which is a plentiful and inexpensive material on Earth [8]. Water, when exposed to high temperatures or radiation, can undergo splitting by thermochemical, photobiological, or photoelectrolytical reactions. Splitting can also occur by photocatalytic conversion of solar energy into chemical energy. A photocatalyst that has been activated by solar radiation splits water into oxygen and hydrogen in one or two steps during the photocatalysis process [9]. Common photocatalysts used for this photocatalytic water splitting include many semiconductors that have been designed specifically for this purpose. However, the efficiency of photocatalytic water splitting is still poor; therefore, highly developed photocatalysts that can overcome obstacles in the water oxidation process are needed. A perfect photocatalyst will have a positively topped valence band, a negative bottom to the conduction band, and a somewhat narrow band gap. The small band gap, in the right place to produce an electron–hole pair for a redox reaction, improves solar energy harvesting. The top of the valence band has a redox potential greater than that of water, and the negative bottom of the conduction band promotes the quick release of hydrogen and the photooxidation reaction [10].

Common photocatalysts used for water splitting include semiconductor metal oxides and sulfides, whose employment accelerates the production of hydrogen [11]. These materials can be incorporated as nanomaterials into advanced composite structures, where they can participate directly in photocatalytic water splitting. They can also indirectly improve the efficacy of existing photocatalysts [8], as the choice of nanomaterial structure and size [12,13] can alter the bandwidth, whereas increasing the surface area-to-volume ratio can lead to charge separation and inhibit hole–electron re-joining [14].

Several photocatalytically active materials, such as metal oxides, sulfides, nitrides, oxysulfides, carbon nitrides, and oxynitrides, have been examined for their ability to produce hydrogen from water [15,16,17,18,19,20,21]. The most widely investigated of these are TiO_2_ and ZnO [22,23]. The free excitons of ZnO have a binding energy of 60 meV, and ZnO has a wide direct bandgap of 3.30–3.37 eV at 300 K [23,24]. These two qualities make this semiconductor an appealing choice for many optical and electrical applications [25]. ZnO has a higher electron mobility than TiO_2_ [26], giving it an increased electronic transfer efficiency while reducing recombination process losses. ZnO has an appropriate band structure and satisfies the thermodynamic requirements for water splitting. As a photocatalyst, it is also inexpensive, non-toxic, and retains a high degree of crystallinity and stability. Although its conversion efficiency may not be as high as that of some metal sulfide semiconductor systems [27], its anisotropic growth and crystallization capabilities make it a desirable alternative to other photocatalysts, such as ZnS, CuS, or other core-shell architectures [28].

The advantages of ZnO nanostructures have, therefore, been taken into consideration in the current study, with the goal of fabricating a photocatalyst and determining the impact of morphological modification on ZnO photoelectrochemical activities. Here, ZnO nanostructures were fabricated using an electrodeposition method. The nanostructures were directly grown on indium tin oxide glass (ITO) glass, and specific capping agents were employed to modify the material throughout the electrodeposition process. The end result was the creation of ZnO nanostructures with a variety of densities and shapes, which were then analyzed using X-ray diffraction (XRD), scanning electron microscopy (SEM), UV-visible spectroscopy (UV-Vis), photoluminescence (PL), and photoelectrochemical (PEC) measurements. Further proof-of-concept studies examined the influence of morphology on the PEC performance of ZnO nanostructures.

## 2. Materials and Methods

### 2.1. Materials and Chemicals

Zinc nitrate hexahydrate (Zn(NO_3_)_2_.6H_2_O), bought from Fisher Scientific (Fair Lawn, NJ, USA), was used for ZnO deposition. Ethylenediamine (EDA) and ammonium fluoride (NH_4_F) were purchased from Sigma-Aldrich (St. Louis, MO, USA). Indium tin oxide glass (ITO) on glass (size: 25 mm × 25 mm, resistivity: 7 ohm/cm^2^), purchased from University Wafer Inc. (Boston, MA, USA), was used for ZnO electrodeposition. Acetone, hydrochloric acid, and nitric acid for cleaning and rinsing the ITO substrate were obtained from Fisher Scientific.

### 2.2. Electrodeposition of ZnO Nanostructures on ITO

ZnO was deposited onto the ITO glass substrate using an electrochemical cell with three electrodes. The working electrode (WE) of the cell was the ITO glass substrate; the counter electrode (CE) was a 2 mm diameter platinum wire, and the reference was an Ag/AgCl wire electrode saturated in 1.0 M KCl.

The ITO glass substrates were cleaned in acetone using an ultrasonic cleaner for 10 min, as described in our previous work [29], prior to electrodeposition of the ZnO nanostructures. The substrates were then washed for 2 min in hydrochloric acid and then in nitric acid, with distilled water used to rinse the substrates between each cleaning procedure.

The electrolyte solution for the ZnO nanostructure electrodeposition was prepared by dissolving Zn (NO_3_)_2_ (50 mM) in water. The ZnO nanostructures were then deposited using a CH1601E potentiostat for 30 min at 70 °C with an applied potential of −1.0 V. The two main capping agents, ammonium fluoride (NH_4_F) and ethylenediamine (EDA), were added to the electrolyte solution at concentrations of 15 mM and 10 mM, respectively, prior to the electrodeposition. The ZnO electrodeposition process parameters are given in Table 1. The careful selection of appropriate concentrations (mM) of capping agents is essential for synthesizing oxide nanostructures with a controlled shape, which maximizes their performance and increases their efficiency in photocatalytic processes. In the present work, we performed a number of experiments to identify the ideal capping agent concentration by exploring a range from 10 mM to 100 mM. Finding the ideal conditions for our samples was accomplished quite well using this trial-and-error method.

### 2.3. Sample Characterisation

The XRD patterns were measured using a modified Bruker-Axs D8 diffractometer with parallel beam optics and a PSD LynxEye silicon strip detector. This instrument utilized an unmonochromated Cu Kα source operated at 40 kV with a 30 mA emission current. The incident beam angle was set at 0.5°, and the angular range of the collected patterns was 10° < 2θ < 65°, with a step size of 0.05° counted at 1 s/step. The surface morphology and film thickness were examined using SEM and a JEOL JSM-6301F field emission microscope at an accelerating voltage of 5 keV. A Transmission Electron Microscope (TEM, JEM-2100 HR, JEOL, Tokyo, Japan) was operated at 200 kV. X-ray photoelectron spectroscopy (XPS) was conducted using a Themo K-Alpha spectrometer equipped with monochromated Al K-alpha radiation, a dual-beam charge compensation system, and a constant pass energy of 50 eV. Survey scans were acquired over the 0–1200 eV range, while high-resolution spectra were obtained for the principal peaks of Zn (2p), O (2p), and C (1s). Optical spectra were obtained using a PerkinElmer Fourier transform Lambda 950 spectrometer over a wavelength range of 300–2500 nm, encompassing the ultraviolet (UV), visible (Vis), and near-infrared (NIR) regions. The spectra were referenced against an air background. The PL spectra from 350–800 nm were recorded using an Edinburgh spectrofluorometer equipped with a maximum average power of 5 mW. The excitation wavelength was 380 nm. The PL spectra were recorded under air at room temperature.

### 2.4. Photoelectrochemical Measurements (PEC)

The PEC measurements for all photoelectrodes were conducted in a three-electrode configuration in 1.0 M KOH electrolyte in deionized water (DI), using an electrochemical workstation (a Metrohm potentiostat) under simulated sunlight (100 mW/cm^2^, AM 1.5 G). The reference electrode was Ag/AgCl in saturated KCl, and the counter electrode was a platinum wire. The illumination intensity was calibrated using a silicon reference cell with an optical meter (Newport, Model 1918-R). The electrochemical impedance spectroscopy (EIS) tests were conducted in the dark at the open circuit potential across a frequency range from 10^5^ to 10^−2^ Hz, with an AC voltage amplitude of 5 mV, using 12 points per decade. Mott–Schottky plots were tested over a potential range of −0.6 to 0.6 V (vs. Ag/AgCl) at a frequency of 1000 Hz.

## 3. Results and Discussion

### 3.1. Characterization of the Fabricated ZnO Nanostructures

**Figure 1:**
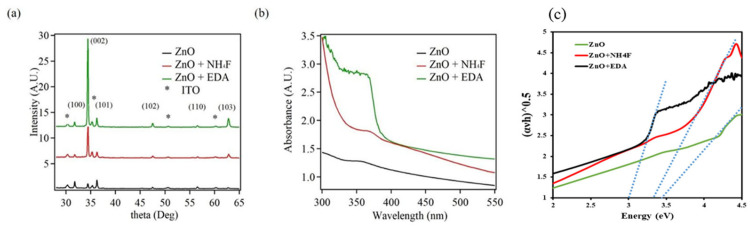
(**a**) X-ray diffraction (XRD) plot, (**b**) UV-vis absorbance spectra and (**c**) the Tauc plot representing the band gap energy of the ZnO, ZnO + NH_4_F, and ZnO + EDA nanostructures. All samples were prepared by electrodeposition method.

The crystalline nature and preferred orientation of the synthesized ZnO nanostructures were examined using XRD. Three samples were prepared for the investigation: unmodified ZnO (abbreviated as ZnO); ZnO nanostructures prepared in the presence of NH_4_F (abbreviated as ZnO + NH_4_F); and ZnO nanostructures prepared in the presence of EDA (abbreviated as ZnO + EDA). The ZnO nanostructures determined by XRD measurements are displayed in Figure 1a. For every sample, the XRD plot shows a crystalline wurtzite structure. The plots and the JCPDS No. 36-1451 reference file were perfectly matched. The ZnO nanostructures showed a preferential orientation with a dominating (002) crystal plane at 2θ = 34.7° in the presence of capping chemicals. The XRD plots of ZnO + NH_4_F and ZnO + EDA clearly showed this preferred orientation and confirmed that the addition of the capping agents changed the ZnO nanostructure morphology.

Figure 1b shows an overview of the ZnO nanostructure absorption characteristics determined from UV-Vis measurements of the ZnO nanostructures. The UV-Vis spectra revealed an absorption peak at 360 nm, corresponding to a bandgap of 3.45 eV. By contrast, the absorption peaks of the ZnO + NH_4_F and ZnO + EDA samples showed a blue shift to 350 nm, with a bandgap shift to 3.35 and 3.0 eV, respectively (Figure 1c), indicating that the presence of the NH_4_F and EDA capping agents changed the ZnO nanostructure morphology and caused the blue shift.

The morphology of the ZnO nanostructure has a direct effect on the UV-vis absorbance spectra because different shapes interact differently with light. For example, nanostructure rods or needles may have a longer effective path length for light absorption due to their anisotropic shape, which can shift or broaden the absorbance peaks. Changes in morphology can also introduce or reduce the number of surface defects or trap states, which then alter the absorption behavior. The nanostructure might also have more surface-related defects than are found on hexagonal rods, which would also lead to different UV absorption characteristics.

However, changes in morphology can also modify the band structure of ZnO, especially due to quantum confinement and surface effects. In materials such as ZnO, the band structure can be anisotropic, meaning that the energy bands vary depending on the crystallographic direction. Morphologies that expose different crystallographic planes will have slightly different effective masses of electrons and holes and will modify how the electrons interact with the lattice. This can lead to subtle changes in the band structure.

**Figure 2:**
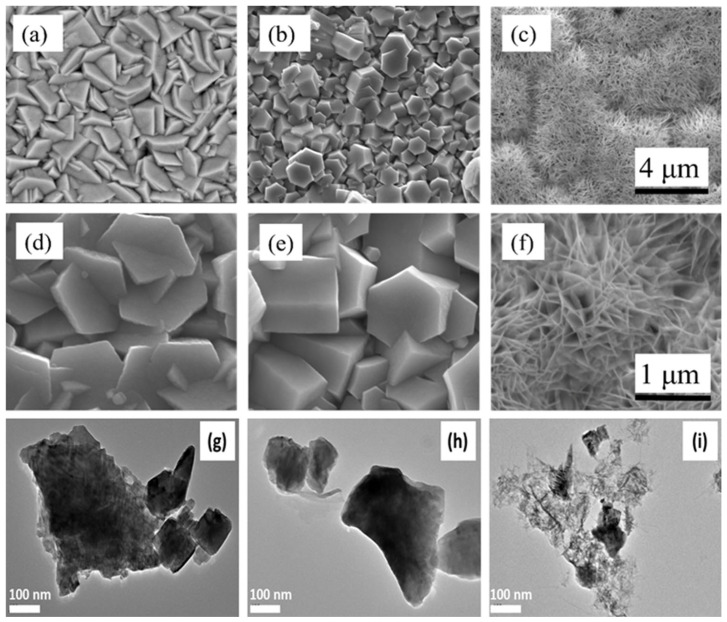
Illustrates SEM images of the surface of (**a**,**d**) ZnO, (**b**,**e**) ZnO + EDA, and (**c**,**f**) ZnO + NH_4_F nanostructure grown by electrodeposition method on ITO glass. TEM images of (**g**) untreated ZnO, (**h**) ZnO + EDA, and (**i**) ZnO + NH_4_F.

The morphological changes in ZnO in the presence of capping agents were confirmed by these characterizations as well as by SEM images of the ZnO nanostructures. Low- and high-magnification SEM images of the ZnO nanostructures are displayed in Figure 2. The surface of the untreated ZnO nanostructure consists of narrow pyramidal features emerging from the substrate Figure 2a,b with diameters of approximately 500 nm. The ZnO + EDA sample featured a hexagonal nanostructure with a diameter of 1.2 μm and a thickness of 295 nm (Figure 2b,e). The presence of EDA in the depositing electrolyte was the reason given for this change in morphology. Figure 2b,e showed that the morphological change was caused by a tendency for the EDA to adsorb onto a specific plane. In comparison to the ZnO reference sample shown in Figure 2a, the hexagonal particles in the ZnO + NH_4_F sample became thinner and smaller, generating a needle-like nanostructure (Figure 2c,f). The needles had a thickness of 50 to 300 nm and a maximum length of 10 μm.

The morphological changes are further confirmed by the TEM results, as presented in Figure 2g–i. The TEM image of the untreated ZnO nanostructure (Figure 2g) reveals narrow pyramidal features. In contrast, the TEM image of ZnO treated with EDA (Figure 2h) shows noticeable aggregation, with particle sizes around 200 nm. Additionally, the TEM image of ZnO treated with NH_4_F (Figure 2i) distinctly exhibits a needle-like nanostructure.

The morphologies of the ZnO + EDA and ZnO + NH_4_F samples differed significantly from that of the reference ZnO sample. The SEM images also verified that the change in morphology during the synthesis of ZnO in the presence of chemical additives was successfully controlled. The different effects of NH_4_F compared to EDA on the morphology of ZnO nanostructures can be attributed to the different chemical interactions of the capping agents with ZnO during the synthesis process. The effect on the ZnO nanostructure morphology is more pronounced for NH_4_F than for EDA because fluoride ions have a direct and stronger influence on the crystal growth direction due to their selective adsorption onto ZnO facets, leading to more distinct morphological changes. The obtained results support the conclusion that the sizes and morphologies of the synthesized ZnO nanostructures are significantly influenced by the capping agents EDA and NH_4_F. The morphology of the zinc oxide was changed, particularly after the addition of NH_4_F, as the nanostructure became smaller and adopted a needle-like shape.

**Figure 3:**
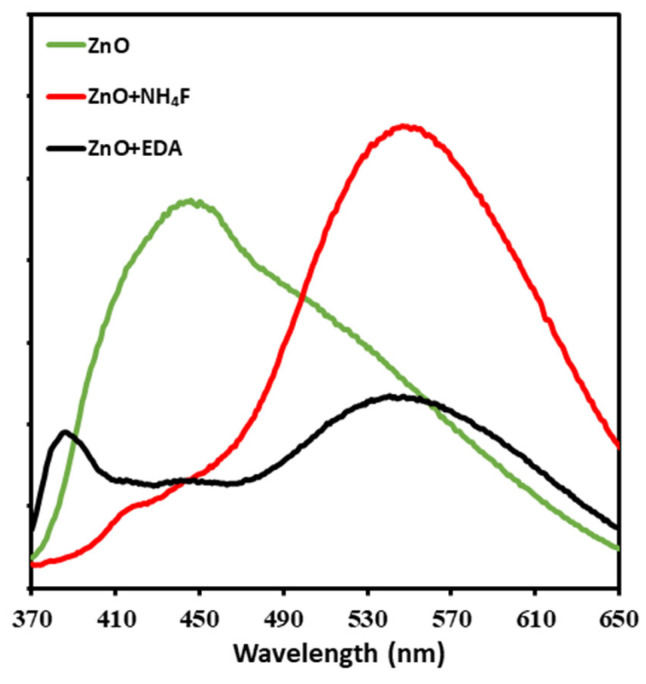
Photoluminescence (PL) measurements of untreated ZnO, ZnO + NH_4_F, and ZnO + EDA films prepared by electrodeposition on ITO glass at 70 °C. The PL emission spectra of ZnO, ZnO + NH_4_F, and ZnO + EDA nanostructures were measured over a wavelength range of 370–650 nm using an excitation wavelength of 380 nm.

Figure 3 shows the PL emissions in the UV−Vis and NIR regions from untreated ZnO nanoparticles and ZnO nanoparticles treated with NH_4_F and EDA. PL spectral emission is a valuable tool for studying charge carrier recombination and investigating the efficiency of charge carrier trapping, migration, separation, and transfer in semiconductors [30,31]. PL emission spectra arise from the radiative recombination of photoexcited carriers, with a higher PL intensity indicating a greater extent of radiative recombination [32]. Figure 3 shows the PL emission spectra of ZnO, ZnO + NH_4_F, and ZnO + EDA nanostructures over a wavelength range of 370–650 nm using an excitation wavelength of 380 nm. All samples showed distinctive PL peaks, with untreated ZnO nanostructures showing a broad peak from 370–570 nm. Interestingly, the PL spectra of ZnO + NH_4_F showed a significantly different peak pattern, with a broad and strong visible emission at 550 nm and a weak band emission at 390 nm. By contrast, the PL spectra of ZnO + EDA exhibited two weaker bands, with emissions centered at 380 nm and 550 nm. The emission band located at 380–390 nm is attributed to near-band emissions, whereas the visible emission peak at 550 nm is attributed to various defects, including oxygen vacancies, zinc vacancies, and oxygen interstitials, which were formed by treating ZnO with the capping agents [33]. However, the changes in the PL spectra of the ZnO nanostructure due to surface morphology alteration arise from the interplay between surface defect passivation and defect state modulation by the capping agents. For instance, oxygen vacancies and zinc interstitials may be common intrinsic defects in ZnO, both of which lead to deep-level emissions in the visible region of the PL spectrum. Capping agents can either reduce these surface-related defects or change their nature, thereby significantly altering the PL spectrum.

**Figure 4:**
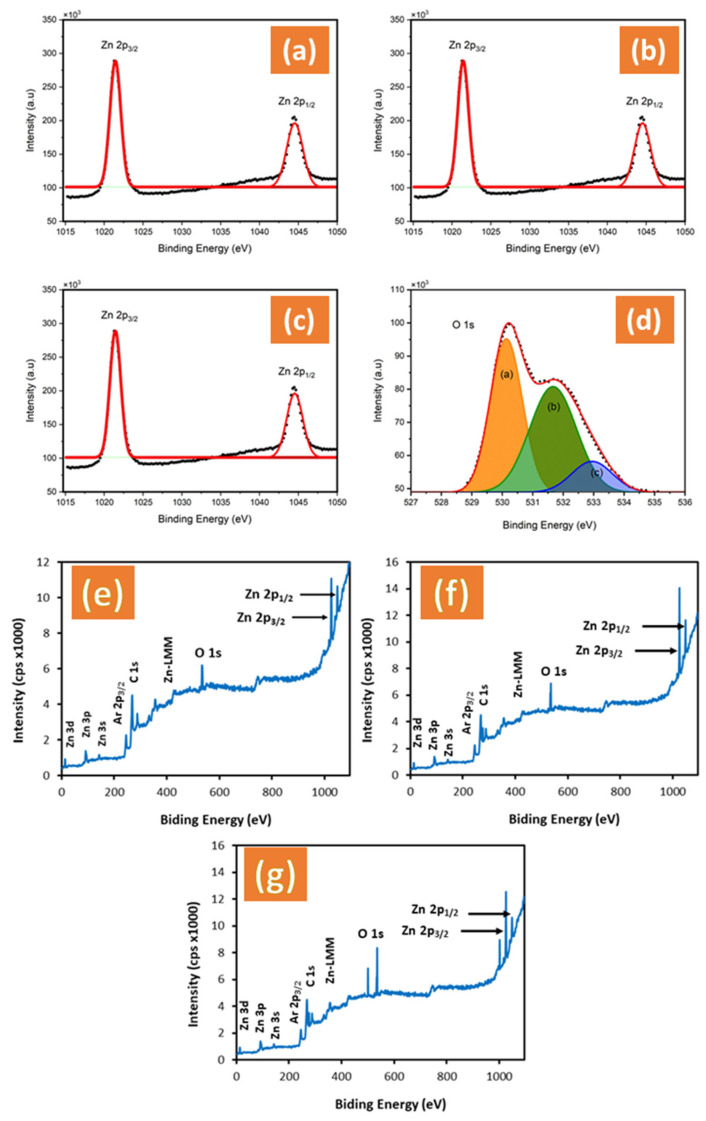
Surface X-ray photoelectron spectroscopy (XPS) spectra of the Zn 2p_1/2_ and 2p_3/2_ core level scans in (**a**) un-treated ZnO film, (**b**) ZnO + NH_4_F, (**c**) ZnO + EDA films, (**d**) O 1s of untreated ZnO, the orange color (a) corresponds to O^2^^−^ ions within the ZnO lattice, the green color (b) attributed to O^−^ and O^2^^−^ ions in oxygen-deficient regions and the blue color (c) is assigned to OH groups on the ZnO surface or adsorbed/dissociated oxygen species, and (**e**–**g**) XPS survey spectrum of untreated ZnO, ZnO + NH_4_F, and ZnO + EDA films.

The oxidation states of all samples on the surfaces were evaluated by XPS. The Zn 2p binding energy region in all samples showed a Zn^2+^ character, with the 2p_3/2_ and 2p_1/2_ peaks centered at 1022.25 and 1045.31 eV, respectively, as shown in Figure 4a–c. The ZnO treated by NH_4_F and ZnO + EDA exhibited no change in oxidation state, as they all showed the 2p_3/2_ and 2p_1/2_ peaks at 1022.25 and 1045.31 eV, respectively, consistent with the Zn^2+^ character [34]. Figure 4d shows the XPS spectra of the O 1s core level for ZnO + NH_4_F, with the 1s peaks centered at 530.0 and 532.00 eV. According to the published reports, these peaks correspond to oxygen at the lattice site and interstitial oxygen, respectively [35]. The deconvolution of the O 1s spectra for the ZnO nanostructure (Figure 4d) indicates broad and asymmetric peaks, reflecting the presence of different oxygen states. Lorentzian–Gaussian curve fitting reveals three distinct components. The first component (a), at a low binding energy of 530.50 eV, corresponds to O^2^^−^ ions within the ZnO lattice. The second component (b), at 532.0 eV, is attributed to O^−^ and O^2^^−^ ions in oxygen-deficient regions, primarily caused by oxygen vacancies. The third component (c), at 533.21 eV, is assigned to OH groups on the ZnO surface or adsorbed/dissociated oxygen species.

Notably, the XPS survey confirmed that zinc and oxygen are the main constituents, with no impurities, including fluorine or nitrogen, detected in the films (Figure 4e–g).

### 3.2. Photoelectrochemical Measurements of ZnO Nanostructures

**Figure 5:**
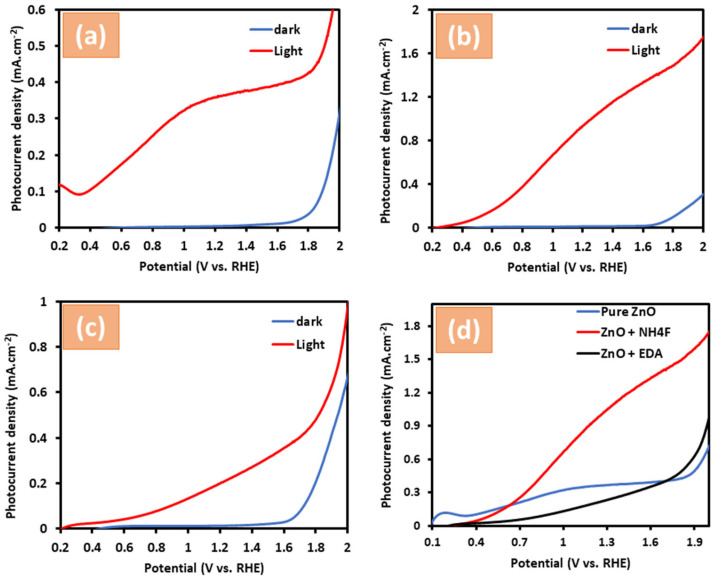
Shows linear sweep voltammetry (LSV) curves of (**a**) untreated ZnO films, (**b**) ZnO + NH_4_F, (**c**) ZnO + EDA, and (**d**) presents a comparison of LSV across all samples. All samples were measured under two conditions: darkness and simulated sunlight (AM 1.5 G, 100 mW/cm^2^) in 1.0 M KOH electrolyte and on ITO glass.

This study explored the influence of NH_4_F and EDA capping agents on the photocatalytic activity of zinc oxide to highlight their potential for improving the use of sunlight as a renewable energy source. As shown in Figure 5, PEC water measurements were conducted in 1.0 M KOH (pH 13.5) to generate linear sweep voltammetry (LSV) curves for untreated ZnO, ZnO capped with NH_4_F and ZnO capped with EDA in darkness and simulated sunlight (AM 1.5 G, 100 mW/cm^2^). Figure 5a shows that under dark conditions, the photocurrent of the untreated ZnO nanostructure sharply increases at a voltage of approximately 1.60 VRHE. However, under simulated sunlight conditions, the same sample exhibited a photocurrent peak of about 0.35 mA cm^−2^ at 1.23 V_RHE_, where RHE corresponds to the potential of the reversible hydrogen electrode. Figure 5b shows the photocurrent graphs of ZnO + NH_4_F under sunlight conditions. Treatment with NH_4_F caused an increase in photocurrent, which reached 1.0 mA cm^−2^ at 1.23 V_RHE_ (Figure 5b). By contrast, ZnO treated with EDA reached a photocurrent of around 0.21 mA cm^−2^ at 1.23 V_RHE_ (Figure 5c), indicating a lower photocurrent for ZnO + EDA than for untreated ZnO. The lower PEC performance for ZnO + EDA could be attributed to its limited stability, as ZnO + EDA exhibited a higher rate of degradation compared to ZnO or ZnO + NH_4_F.

For all samples, the current curves in dark conditions were increased by up to ~1.60 V_RHE_. Figure 5d, which presents a comparison of photocurrent measurements across all samples, reveals that ZnO + NH_4_F exhibited the highest photocurrent at 1.23 V_RHE_. One point to consider is that the enhancement in PEC performance can be attributed to the influence of NH_4_F addition on the morphological changes in the ZnO + NH_4_F nanostructure. This is supported by the SEM images, which revealed a change in morphology in the form of smaller nanostructures that adopted a needle-like shape. Previous reports in the literature have indicated that changes in the morphology of ZnO play a crucial role in enhancing photocurrent density and stability. This enhancement is due to its effects on charge transfer from the electrolyte to the ZnO surface, as well as on carrier concentration and conductivity. Additionally, treating metal oxide surfaces with inorganic acids has been found to increase the presence of hydroxide groups on the surfaces. These hydroxide groups enhance light absorption and introduce reactive sites on the metal oxide surface [36,37]. The solar-to-hydrogen (STH) conversion efficiencies of the photoelectrodes were determined using the following equation [38]:

(1) $$\eta_{\mathrm{STH}}=\frac{j_{ph\times 1.23V}}{P_{in}},$$

where *j_ph_* is the photocurrent density of the system; 1.23 V represents the theoretical minimum voltage needed for water splitting, and *P_in_* is the incident light power, standardized at 100 mW/cm cm^−2^ under the AM 1.5 G spectrum with an intensity of 1 sun. Remarkably, the untreated ZnO photoelectrode achieved a maximum photocurrent of 0.35 mA/ cm^−2^ at 1.23 V vs. RHE, which corresponds to an STH efficiency of 0.40%. In comparison, the STH efficiency of a ZnO + NH_4_F photoelectrode thin film increased to 1.2%, a threefold improvement over the untreated ZnO thin films.

### 3.3. Electrochemical Impedance Spectroscopy (EIS)

EIS is a powerful characterization technique utilized in various fields, including energy, electrocatalysis, and medicine. The use of EIS is particularly appealing for two reasons. One is that EIS data can be employed to determine the physical characteristics, such as diffusion coefficients, chemical reaction rates, and microstructural properties, of the electrochemical (EC) system under investigation. The other is that setting up an EIS experiment is straightforward. Unlike standard EC techniques, which evaluate the relationship between the current and applied potential difference in the time domain, EIS operates uniquely in the frequency domain. The EIS approach calculates the transfer function between the current and potential. To measure EIS, an EC system is subjected to a small (preferably infinitesimal) sinusoidal voltage (in potentiostatic mode) or current (in galvanostatic mode) disturbance across a range of frequencies while simultaneously recording the corresponding sinusoidal current or voltage response. From this, a complex-valued function Z(f) is obtained. This function depends on the perturbation frequency (f) through the voltage-to-current amplitude ratio and phase lag between input and output.

An important point to note is that to be useful, EIS must satisfy the conditions of stability, linearity, and causality. The EC system must be stable, as any variation over time will affect impedance frequency values. The perturbation must also be linear because the application of a sinusoidal disturbance with a sufficiently small amplitude will generate a response that is a sinusoid with the same frequency. However, a large amplitude of the sinusoidal input at a given frequency will result in a measured response that consists of superimposed sinusoids at frequencies f, 2f, and 3f. The third requirement of causality indicates that the measured current (or voltage) must be a direct consequence of the applied voltage (or current) perturbation. Overall, EIS offers valuable insights into the physical and microstructural characteristics of EC systems, while its experimental setup is relatively straightforward compared to other EC techniques [39,40].

**Figure 6:**
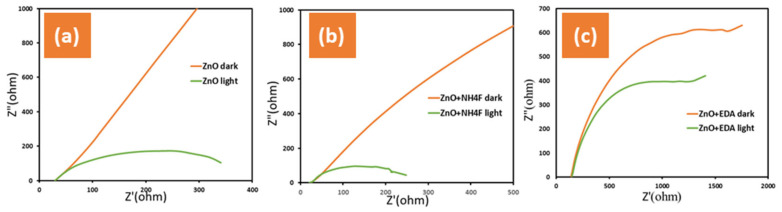
Shows electrochemical impedance spectroscopy (EIS) measured under two conditions: darkness and simulated sunlight (AM 1.5 G, 100 mW/cm^2^) in 1.0 M KOH electrolyte and on ITO glass for (**a**) untreated ZnO, (**b**) ZnO + NH_4_F, and (**c**) ZnO + EDA films.

The EIS measurements were employed here to analyze the interfacial properties between the photoanode and electrolyte of the fabricated samples. The Nyquist plots, representing the imaginary and real components of the EIS plots (Z’ versus -Z’’), were measured at 1.0 VRHE under dark and simulated sunlight conditions (AM 1.5 G, 100 mW/cm^2^) (Figure 6). The EIS, employed to determine the solution resistance and charge transfer resistance, revealed that the untreated ZnO + NH_4_F films had the lowest R_ct_ values in the dark and simulated sunlight (Figure 6a), whereas the untreated ZnO + EDA films exhibited the highest R_ct_ values under both conditions (Figure 6c). Higher R_ct_ for ZnO + EDA compared to pure ZnO or ZnO + NH_4_F indicates that the addition of EDA does not enhance and may even hinder charge transfer. This contrasts with ZnO + NH_4_F, where the addition of NH_4_F during preparation improved the morphology and lowered the R_ct_. value. The increase in R_ct_ for ZnO + EDA could be linked to the creation of an unfavorable morphology or the blocking of active sites, which would lead to poorer electrochemical performance and stability. Furthermore, ZnO + EDA exhibited poor stability, suggesting that degradation may have occurred during the measurements.

In the present study, R_ct_ represents the charge transfer resistance across the film–electrolyte interface and is attributed to the excellent conductivity of the film. An inverse relationship is known to exist between impedance and conductivity, and under light conditions, the conductivity of the ZnO + NH_4_F samples is stronger than that of the other samples in the 1.0 M KOH electrolyte, resulting in lower impedance. The addition of NH_4_F to the samples during the deposition is believed to enhance the conductivity of the ZnO + NH_4_F films by reducing the recombination of electron–hole pairs, thus improving the photocurrent density. Overall, the ZnO + NH_4_F films demonstrated the best performance in terms of ionic and electronic transfer rates.

The changes in the morphology of the ZnO nanostructure directly influence the impedance response by altering the charge transfer resistance, capacitance, and ion diffusion properties. These changes are reflected in the Nyquist plots obtained from the EIS measurements. A thinner and smaller morphology, as we can see from the SEM image of ZnO + NH_4_F, results in lower impedance and better electrochemical performance due to the increased surface area, which can increase the number of active sites available for charge transfer. This reduces the charge transfer resistance, resulting in a smaller semicircle in the Nyquist plot.

**Figure 7:**
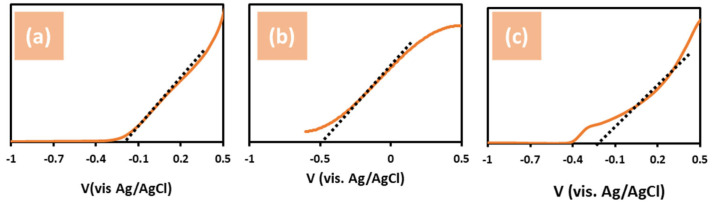
Mott−Schottky plots for three photoelectrodes in 1.0 M KOH and under simulated solar illumination (100 mW/cm^2^), using (**a**) un-treated ZnO, (**b**) ZnO + NH_4_F, and (**c**) ZnO + EDA nanostructure film. Flat band potential of the samples was calculated for this analysis.

Mott–Schottky (M–S) calculations were used to investigate the flat-band potential (*V_fb_*) and donor density (*N_D_*) of untreated ZnO, ZnO + NH_4_F, and ZnO + EDA nanostructures. Measurements were obtained at a frequency of 1.0 kHz using the following equation [41,42,43]:

(2) $$\frac{1}{C^{2}}=\frac{2}{\varepsilon \varepsilon_{ο}eN_{D}A^{2}}\left(E-V_{fb}-\frac{k_{B}T}{e}\right),$$

where *A* is the active area; *e* is the electron charge; *ε_ο_* is the permittivity of a vacuum; *ε* is the dielectric constant; *T* is the absolute temperature; *k_B_* is the Boltzmann constant, and *E* is the applied potential. The Mott–Schottky plots shown in Figure 7 exhibited the n-type conductivity of all samples. Figure 6a shows that the untreated ZnO nanostructure reveals a flat band gap potential at -2.1 V_Ag/AgCl_, whereas the flat band gaps of the ZnO treated with NH_4_F and EDA are calculated as −5.0 and −2.5 V_Ag/AgCl_, respectively (Figure 7b,c). By comparison, ZnO + NH_4_F displayed the highest negative flat band potential (V_fb_) value. However, since the flat band potential (V_fb_) of ZnO is typically around −0.5 V vs. Ag/AgCl, our results for ZnO are generally consistent with those reported in the literature [44,45,46].

A high negative flat band potential *V_fb_* indicates a significant space charge region potential. This is crucial for photocatalytic activity in hydrogen reduction reactions, as the elevated flat band potential of the semiconductor provides a strong driving force to separate photogenerated electron–hole pairs in this region [30,47,48]. Overall, the changes in ZnO morphology directly influenced the M–S measurements by modifying key properties, such as carrier density, capacitance, and flat-band potential. Smoother, thinner morphologies in ZnO + NH_4_F lead to higher capacitance and lower charge transfer resistance, whereas larger particles increase carrier trapping, leading to reduced carrier density and shifts in the flat-band potential.

**Figure 8:**
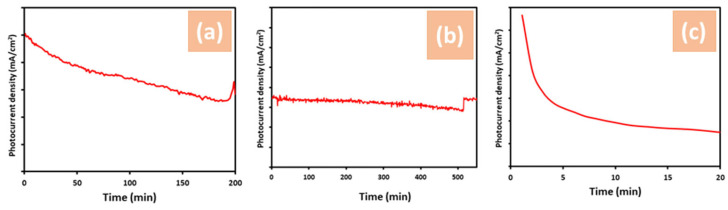
The stability of (**a**) untreated ZnO, (**b**) ZnO + NH_4_F, and (**c**) ZnO + EDA nanostructure films under simulated sunlight with an intensity of 100 mW/cm^2^ and photocurrent density of approximately 1.5 mA/cm^2^ in 1.0 M KOH electrolyte.

The stability of photoelectrodes is crucial for the generation and commercial viability of PEC hydrogen production devices. Consequently, methods to enhance the stability of semiconductors have garnered significant attention in recent studies. Figure 7 illustrates the stability of ZnO, ZnO + NH_4_F, and ZnO + EDA nanostructures under simulated sunlight with an intensity of 100 mW/cm^2^. The ZnO + NH_4_F remained stable for over 550 min (Figure 8b), maintaining a photocurrent density of approximately 1.5 mA/cm^2^. The ZnO + EDA nanostructure exhibited the lowest stability. As shown in Figure 8c, degradation of the film on ITO glass occurred immediately. The untreated ZnO also remained stable for less than 200 min (Figure 8a). Overall, adding NH_4_F during the preparation of ZnO nanostructures could clearly improve stability by enhancing adhesion to substrates and producing a more refined morphology. This improved adhesion and uniformity reduced the chances of material peeling or detaching during long-term operation.

**Figure 9:**
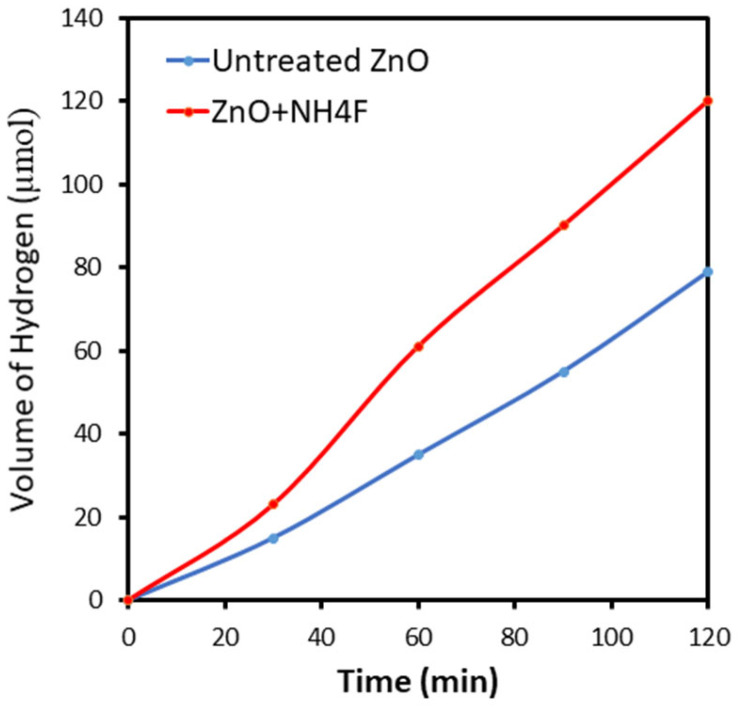
Gas evolution of hydrogen and oxygen from the untreated ZnO and ZnO + NH_4_F anode measured by gas chromatography (GC).

The direct generation of the photocurrent from water splitting, rather than from side reactions, was verified by measuring the photogenerated hydrogen using gas chromatography while applying a constant potential of 1.2 V vs. RHE. Hydrogen evolution from the sample anode was monitored at regular intervals. After 120 min of reaction, 79 and 120 μmol of hydrogen were detected for the untreated ZnO and ZnO + NH_4_F anodes, respectively (Figure 9). This indicated a greater hydrogen evolution by ZnO + NH_4_F than by untreated ZnO, which can be attributed to surface modification and improved charge transfer. This result suggests that under an applied bias of 1.2 V vis. RHE, the photogenerated holes are able to migrate from the valence band of untreated and treated ZnO to the electrolyte.

**Figure 10:**
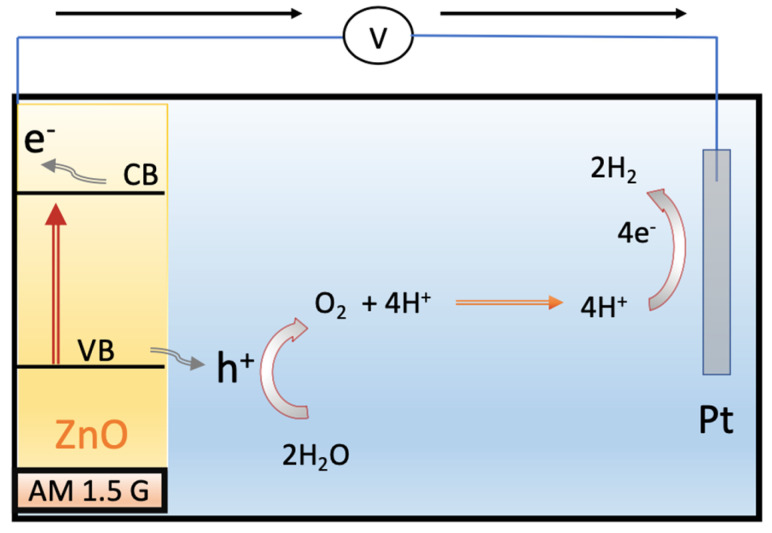
Schematic diagrams depicting the photoelectrochemical (PEC) water splitting mechanism of ZnO nanostructure on an ITO substrate.

Figure 10 illustrates the proposed mechanism for photoelectrochemical (PEC) water splitting using a ZnO photoanode under visible light illumination. The valence band (VB) position of ZnO is estimated as 2.92 eV, while the conduction band (CB) position is around −0.33 eV, according to a previous report [49]. The VB position of ZnO is more positive than the oxidation potential (1.23 eV vs. RHE), indicating that holes generated through photoexcitation in the valence band have sufficient potential to oxidize water. Concurrently, electrons are transferred from the conduction band of ZnO to the cathode, leading to water reduction. The morphological changes in ZnO enhance charge transport, thereby reducing the electron–hole recombination rate and improving the efficiency of electron–hole separation.

## 4. Conclusions

This paper shows the preparation of films by electrodeposition on ITO glass using untreated ZnO, ZnO treated with NH_4_F, and ZnO treated with EDA for spontaneous water splitting. The addition of a certain amount of NH_4_F during the deposition process significantly changed the morphology of the ZnO nanostructure, as shown in Figure 2. The film prepared with ZnO treated with NH_4_F showed a significant increase in photocurrent compared to the untreated ZnO film in a 1.0 M KOH electrolyte, as noted by the PEC water-splitting measurements. This improvement in the photocurrent was attributed to the impact of NH_4_F on ZnO morphology. PL spectra investigations revealed that the enhancement in PEC performance was correlated with variations in the intensity of the PL spectra peaks.

These results are very promising and indicate exciting potential for developing advanced photocatalytic materials for green hydrogen production and clean energy. The successful application of the NH_4_F treatment to enhance the PEC performance of ZnO nanostructures could open the door to further enhancements in water-splitting efficiency and the advancement of sustainable energy technologies.

## Figures and Tables

**Table 1:** Process parameters used for the fabrication of the ZnO nanostructures.

Process Parameters	ZnO	ZnO + NH_4_F	ZnO + EDA
(Zn (NO_3_)_2_ (mM)	50	50	50
NH_4_F (mM)	0.0	15	0.0
EDA (mM)	0.0	0.0	10
Temp (°C)	70	70	70
Time (min)	30	30	30
Potential (V)	−1.0	−1.0	−1.0

## Data Availability

The original contributions presented in the study are included in the article, further inquiries can be directed to the corresponding authors.
